# Endocrine disrupting potency and toxicity of novel sophorolipid quaternary ammonium salts

**DOI:** 10.1007/s10646-021-02378-6

**Published:** 2021-03-17

**Authors:** Ewa Liwarska-Bizukojc, Christian V. Stevens, Elisabeth I. P. Delbeke, Kevin M. Van Geem

**Affiliations:** 1 Institute of Environmental Engineering and Building Installations, Lodz University of Technology, Al. Politechniki 6, 90-924 Lodz, Poland; 2 Faculty of Bioscience Engineering, Department of Green Chemistry and Technology, Ghent University, Coupure Links 653, B-9000 Ghent, Belgium; 3 Faculty of Engineering and Architecture, Department of Materials, Textile and Chemical Engineering, Ghent University, Technologiepark 914, B-9052 Ghent-Zwijnaarde, Belgium

**Keywords:** Endocrine disrupting activity, Micropollutants, Quaternary ammonium sophorolipids, Toxicity, Yeast assay

## Abstract

A new class of biosurfactants, namely quaternary ammonium sophorolipids (SQAS), suitable for pharmaceutical applications, was tested for the evaluation of their (anti)estrogenic and (anti)androgenic potency with the help of YES/YAS assays. Also their toxicity towards yeasts (*Saccharomyces cerevisiae*) and bacteria (*Escherichia coli*) was checked. The results achieved for SQAS, which can be regarded as potential micropollutants, were compared with those obtained for two well-known micropollutants diclofenac and 17α-ethinylestradiol subjected to the same testing procedures. This work demonstrated that acetylation of the hydroxyl group of the carbohydrate head of SQAS decreased the toxicity of this class of biosurfactants towards *Saccharomyces cerevisiae*. Furthermore, it contributed to the decrease of their endocrine disrupting potency. None of the SQAS studied showed clear agonist activity for female or male hormones. SQAS1 and SQAS2 revealed weak antiestrogenic and antiandrogenic potency. All of these properties were weaker, not only to the potency of the appropriate positive control in the antagonists bioassays, but also compared to the potency of other tested compounds, i.e. DCF and EE2. SQAS3 possessed most probably inhibitory activity towards male hormones. Moreover, cytotoxicity of two out of four studied SQAS at the highest concentrations towards the strains of *Saccharomyces cerevisiae* interfered with the endocrine disruption activity. It would be also worth studying it with the use of another endocrine activity test.

## Introduction

Some natural and synthetic compounds that are able to interfere with the usual functioning of the endocrine system in humans and animals are collectively called endocrine disrupting compounds (EDCs). This group of chemicals mainly consists of natural and synthetic hormones and their metabolites, but also several non-steroidal synthetic compounds that are used as plasticizers, flame retardants, surfactants and pesticides as well as some pharmaceutical and personal care products (Caliman and Gavrilescu 2009; Hamid and Eskicioglu 2012). Most of them end up in the sewage system after use and finally reach the wastewater treatment plants (WWTPs). Their concentrations in the influent of WWTPs range usually between 0.1 and 10 μg l^−1^, so that these chemicals are called micropollutants (Luo et al. 2014). A major contributor to the total estrogenicity of municipal wastewater is the synthetic estrogen 17α-ethinylestradiol (EE2), which is widely used in contraceptive pills. Apart from EE2, the highest concentrations in raw municipal wastewater were reported for potential non-steroidal synthetic EDCs such as nonylphenol and diclofenac (Janex–Habibi et al. 2009; Rosal et al. 2010). For example, the reported concentration of diclofenac varied from below 0.001 to 94.2 μg l^−1^ (Kasprzyk–Hordern et al. 2009; Stamatis and Konstantinou 2013; Ruel et al. 2012; Loos et al. 2013; Gao et al. 2014). The fate of micropollutants in WWTPs depends on the individual physicochemical and biological properties of each compound. Ruel et al. (2012) estimated that on average 80% of the load of micropollutants was removed by conventional activated sludge systems in WWTPs. Micropollutants were usually removed from wastewater as a result of sorption and/or microbiological degradation. Sorption onto suspended colloids and particles caused to the removal of these chemicals via waste sludge. According to the classification proposed by Luo et al (2014) human estrogens, EE2, bisphenol A and triclosan were highly removed from wastewater (degree above 70%); nonylphenol could be classified as moderately removed (degree from 40 to 70%), while atrazine and diclofenac were poorly removed (degree below 40%).

The number of chemicals introduced on the market is still increasing and some of them are classified as environmentally relevant emerging contaminants (Richardson and Ternes 2018). Therefore, it is required to evaluate their physical, chemical and biological properties at the early stage of their life, i.e. before they go into widespread use. Due to thorough studies on chemical properties, it is easier to predict their fate and behaviour in the environment and to avoid threats to living organisms including humans. One of the new classes of chemicals particularly requiring a full description of biological properties, is the class of biosurfactants, namely sophorolipid quaternary ammonium salts.

Sophorolipids are extracellular biosurfactants mainly biosynthesized by yeasts such as *Candida bombicola*, *Candida apicola* and *Starmerella bombicola*. They possess high surface activity, low foaming ability, fast wetting action and low toxicity. Furthermore, they can be completely biodegradable. All these features make sophorolipids an alternative to petrochemical-based surfactants. Nevertheless, their application as detergents is limited due to higher production costs compared to synthetic surfactants. Therefore, other application areas of sophorolipids are looked for.

Sophorolipids, particularly lactonic sophorolipids exhibit antimicrobial activity towards Gram-positive and Gram-negative bacteria (Shah and Badia 2007). Such sophorolipid derivatives as sophorolipids diacetate ethyl esters were reported as the most potent spermicidal and virucidal agents out of those studied by Shah et al. (2005). They also exhibit cytotoxic effects on selected cancer cells (Jing et al. 2006). Custom-built biosurfactants with improved performance, suitable for biomedical applications can be synthesized by chemical and enzymatic modification (Sajna et al. 2015; Delbeke et al. 2016).

An innovative modification pathway of sophorolipids has been described by the Stevens group (Ghent University, Belgium) leading to a new class of biosurfactants, i.e. quaternary ammonium sophorolipids (SQAS). It occurred that some of the new synthesized compounds were more active against bacteria than the antibiotic gentamicin sulfate.

In this work, four SQAS of different chemical composition synthesized by Delbeke et al. (2015) were tested. The criterion of the selection of SQAS was the value of the minimum inhibitory concentration (MIC) determined in tests with Gram-positive and Gram-negative bacteria. The selected SQAS are characterized by minimum inhibitory concentrations at the same or higher level than that of gentamicin.

The main aim of this work was to determine the (anti)estrogenic and (anti)androgenic properties of the representatives of the innovative class of biosurfactants (SQAS), and to compare the results with those obtained for the well-known micropollutants (diclofenac and 17α-ethinylestradiol). Additionally, the toxicity of all tested compounds towards the pure culture of *Escherichia coli* was checked.

## Materials and methods

### Compounds tested

Four innovative SQAS and two common micropollutants were subjected to tests to determine their endocrine potency and toxicity in this study. SQAS were synthesized at the Department of Green Chemistry and Technology (Ghent University, Belgium). As described in the introduction, the MIC value was assumed as the criterion for the selection of SQAS. The synthesis method and physicochemical properties of the SQAS tested as well as their potential applications were described elsewhere (Delbeke et al. 2015; Delbeke et al. 2016). In view of micropollutant behavior, the compounds were compared to diclofenac and 17α-ethinylestradiol. They were purchased from Sigma-Aldrich (Sigma-Aldrich, Germany) and their purity was not lower than 98%.

Chemical names and structures as well as the codes of all compounds studied are shown in Table 1.

**Table 1:** Characteristics of the compounds tested

Chemical name	Mole-cular weight (g mol^−1^)	Code	Chemical structure
N,N-dimethyl,N-octadecyl-(8-L-[(2″,3′,3″,4′,4″,6′,6″-heptaacetoxy-2′-O-β-D-glucopyranosyl-β-D-glucopyranosyl)-oxy])nonan-1 ammonium iodide	1185	SQAS1	
N-benzyl,N-methyl,N-octadecyl-(8-L-[(2″,3′,3″,4′,4″,6′,6″ heptaacetoxy-2′-O-β-D glucopyranosyl-β-D-glucopyranosyl)-oxy])nonan-1-ammonium iodide	1228	SQAS2	
N,N-dimethyl,N-octadecyl-(8-L-[(2β-O-β-D-glucopyranosyl-β-D-glucopyranosyl)-oxy])nonan-1-ammonium iodide	891	SQAS3	
N-benzyl,N-methyl,N-octadecyl-(8-L-[(2β-O-β-D-glucopyranosyl-β-D-glucopyranosyl)-oxy])nonan-1-ammonium iodide	933	SQAS4	
[2-(2,6-Dichloroanilin) phenyl]acetic acid	296.16	DCF	
17α-ethinylestradiol	296.40	EE2	

### XenoScreen YES/YAS^TM^ test

The XenoScreen YES/YAS^TM^ test, i.e. the Yeast Estrogen Screen (YES)/Yeast Androgen Screen (YAS), was used for the determination of the estrogenic, antiestrogenic, androgenic and antiandrogenic potency of the tested compounds. This test is based on the use of genetically modified strains of *Saccharomyces cerevisiae* with DNA sequences of human estrogen receptor (hERα) or human androgen receptor (hAR) that were stably incorporated into the yeast genome (Routledge and Sumpter 1996). Additionally, the cells contain an expression plasmid carrying the reporter gene lacZ encoding for the enzyme β-galactosidase and estrogen (YES) or androgen (YAS) responsive elements (Routledge and Sumpter 1996; Sohoni and Sumpter 1998).

The activation of the hormone receptors (hERα or hAR) by the binding of an endocrine active substance leads to the expression of β-galactosidase. The β-galactosidase is secreted into the medium and converts the yellow substrate chlorophenol red-β-D-galactopyranoside (CPRG) into a red product, which can be quantified colorimetrically at 570 nm. Simultaneously, the assay identifies the cytotoxic effects of the tested compounds on yeast cells. Cytotoxic effects lead to growth arrest or lysis of the yeast cells used in the tests. It is measured as a reduction of light scatter at 690 nm.

The test was carried out according to the instruction delivered by the manufacturer Xenometrix AG (Switzerland). It consisted of six main steps: (1) yeast cultures preparation, (2) preparations and control experiments (17-β estradiol (E2) for YES agonists, 4-hydroxytamoxifen (HT) for YES antagonists, 5α-dihydrotestosterone (DHT) for YAS agonists and flutamide (FL) for YAS antagonists) and their transfer to the assay plates, (3) transfer of YES and YAS yeast cells to the assay plates, (4) incubation of the assay plates for 48 h at 32 °C in the presence of the substrate for β-galactosidase on a rotating platform of the orbital shaker MB100-4A at approximately 100 rpm, (5) reading the assay plates using a microplate photospectrometer MB-530 at 570 and 690 nm, (6) calculation and interpretation of the results towards (anti)estrogenic and (anti)androgenic activity of the tested samples and their cytotoxicity. The individual stock solution for each tested compound in deionized water at the concentration of 10^−2^ M was prepared. Then, it was added to the wells in order to achieve the following final concentrations: 3.16·10^−8^, 1·10^−7^, 3.16·10^−7^, 1·10^−6^, 3.16·10^−6^, 1·10^−5^, 3.16·10^−5^ and 1·10^−4^ M. Each bioassay was repeated four times.

Based upon the measurement of the absorbance, the growth factor (G) and induction (I_R_) ratio were calculated from the following equations.

1 $$G = \frac{{A_{690,S}}}{{A_{690,N}}}$$

2 $$I_R = \frac{1}{G} \cdot \frac{{\left( {A_{570,S} - A_{690,S}} \right)}}{{\left( {A_{570,N} - A_{690,S}} \right)}}$$

where A_690,S_ and A_570,S_ are the absorbances of samples respectively at 690 and 570 nm and A_690,N_ and A_570,N_ are the absorbances of the solvent control respectively at 690 and 570 nm.

The growth factor is regarded as the endpoint in the evaluation of cytotoxicity. The value of G less or equal to 0.5 indicates on possible cytotoxic effects of the compound tested to the yeast cells.

In order to evaluate the results, the criterion was adopted that the tested sample has agonistic YES/YAS properties if the value of I_R_ ≥ 1.5 (for control solutions) and has antagonistic YES/YAS properties if the value of I_R_ ≤ 66.7 % of the value obtained for the control sample (Szczepańska et al. 2018). It was called the IR criterion in this work.

The values of estrogen equivalent (EEQ) and androgen equivalent (AEQ) were calculated. They correspond to the concentration of E2 and DHT, respectively, which would give the same activity as the sample tested. The calculations were made if the equivalent concentration was not lower than 10^−10^ M in the YES tests or 10^−9^ M in the YAS tests. For antiestrogenic and antiandrogenic potency, these equivalent concentrations were called aEEQ or aAEQ, respectively.

Additionally, the values of the effective concentrations (EC50) were determined. In the agonist assays EC50 means the effective concentration at 50% of I_R,MAX_, where I_R,MAX_ is the highest I_R_ of the compound at a non-toxic concentration. At the same time in the antagonist assay, the value of EC50 was calculated only if the percentage of I_R,MIN_ relative to the fitted I_R_ was equal or lower than 50%, where I_R,MIN_ is the lowest I_R_ at the non-toxic concentration of the compound tested.

### Determination of toxicity towards *Escherichia coli*

In order to determine the toxicity of the compounds tested towards bacteria, the ToxTrak^TM^ method was applied. This method is based on the reduction of resazurin, a redox-active dye, by bacterial respiration. When it is reduced, resazurin changes color from blue to pink. Toxic substances can inhibit the rate of resazurin reduction, what can be measured colorimetrically. The endpoint of the ToxTrak^TM^ method is the inhibition of the bacterial respirometric activity expressed as the degree of inhibition (%). In agreement with the guidelines of the manufacturer, it was assumed that the degree of inhibition equal to 10% correlates with the Lowest Observable Effect Concentration (LOEC).

In this work, the ToxTrak^TM^ test was used to measure the toxicity of the compounds tested towards the pure culture of bacteria *Escherichia coli* DSM 30083. Each of the compounds was tested individually. The absorbance was measured with the use of a spectrophotometer DR 6000 at λ = 603 nm. The test was made in accordance to the guidelines for ToxTrak^TM^ (Toxicity ToxTrak^TM^ Method 10017, HACH LANGE manual. https://www.hach.com/toxtrak-reagent-pk-50-powder-pillows/product-parameter-reagent?id=7640273472&callback=qs). Each sample was analyzed in four replicates.

## Results and discussion

In this work (anti)estrogenic and (anti)androgenic properties of the chemicals were evaluated with the use of two criteria. These were the EC50 values (Table 2) and the relation between the I_R_ of the tested sample and the control sample, together with the values of the equivalent concentrations (Table 3). If any compound revealed agonistic or antagonistic endocrine activity according to the IR criterion it was indicated by the sign “+” in Table 3. If not, the sign “–” was used.

**Table 2:** Values of the effective concentration (EC50) assessed for the compounds tested including the control substances

Type of endocrine activity	Compound	EC50 [M]	*R*^2^
YES agonist	17ß-estradiol (E2)	2.12·10^−10^	0.978
	SQAS1	n/d	n/d
	SQAS2	n/d	n/d
	SQAS3	n/d	n/d
	SQAS4	n/d	n/d
	DCF	1.50·10^−4a^	0.998
	EE2	4.40·10^−6^	0.906
YAS agonist	5α-dihydrotestosterone (DHT)	8.02·10^−9^	0.985
	SQAS1	n/d	n/d
	SQAS2	n/d	n/d
	SQAS3	n/d	n/d
	SQAS4	n/d	n/d
	DCF	n/d	n/d
	EE2	n/d	n/d
YES antagonist	4-Hydroxytamoxifen (HT)	6.92·10^−8^	0.994
	SQAS1	9.70·10^−5a^	0.663
	SQAS2	1.70·10^−4a^	0.704
	SQAS3	n/d	n/d
	SQAS4	n/d	n/d
	DCF	3.00·10^−7^	0.729
	EE2	6.50·10^−7^	0.802
YAS antagonist	flutamide (FL)	6.78·10^−6^	0.966
	SQAS1	5.10·10^−5a^	0.997
	SQAS2	1.70·10^−4a^	0.722
	SQAS3	7.90·10^−7a^	0.691
	SQAS4	n/d	n/d
	DCF	7.5·10^−8a^	0.702
	EE2	1.3·10^−6a^	0.710

^a^The extrapolated values

**Table 3:** (Anti)estrogenic and (anti)androgenic activities of the compounds tested based upon the IR criterion. The values of the equivalent concentrations

Compound	Estrogenic activity				Androgenic activity			
	Agonistic estrogenic activity		Antagonistic estrogenic activity		Agonistic androgenic activity		Antagonistic androgenic activity	
	I_R_ criterion	EEQ [M]	I_R_ criterion	aEEQ [M]	I_R_ criterion	AEQ [M]	I_R_ criterion	aAEQ [M]
SQAS1	–	<10^−10^	–	8.67·10^−4^	–	<10^−9^	–	<10^−9^
SQAS2	–	<10^−10^	–	5.1·10^−4^	–	<10^−9^	–	8.23·10^−2^ ± 0.02
SQAS3	–	<10^−10^	–	<10^−10^	+	<10^−9^	+	<10^−9^
SQAS4	–	<10^−10^	–	<10^−10^	+	<10^−9^	–	<10^−9^
DCF	+	1.19·10^−6^	+	1.8·10^−1^ ± 0.25	–	<10^−9^	+	<10^−9^
EE2	+	3.9·10^−5^ ± 0.82·10^−5^	+	8.3·10^−2^ ± 0.14	–	<10^−9^	+	5.29 ± 5.10

“+” indicates on agonistic or antagonistic endocrine activity according to the I_R_ criterion; “−” indicates on neither agonistic nor antagonistic endocrine activity according to the I_R_ criterion

### Estrogenic and androgenic activities

It was found that none of the newly synthesized biosurfactants (SQAS) exhibited agonistic properties in relation to either estrogenic or androgenic hormonal activities (Tables 2 and 3). In the case of two SQAS, namely SQAS1 and SQAS2 this was confirmed by both criteria used in this work (Tables 2 and 3). At the same time the other two biosurfactants (SQAS3 and SQAS4) showed agonistic properties related to the male hormone according to the IR criterion (Table 3). However, the values of equivalent concentrations (AEQ) could not be determined for SQAS3 and SQAS4 with respect to their agonistic androgen activity. The problems with the determination of the equivalent concentrations and the values of EC50 were most probably connected with the cytotoxicity of SQAS3 and SQAS4 towards YAS yeast cells at the two highest concentrations tested. The toxicity trumped the endocrine disruption activity, and therefore it is difficult to judge, whether SQAS3 and SQAS4 can be classified as androgen agonists.

The cytotoxicity of SQAS also contributed to some problems to achieve the highest degrees of correlation of the experimental points to the sigmoidal dose-response curve. The values of the degree of correlation (*R*^2^) were usually lower in the experiments with SQAS than in these with DCF or EE2 (Table 2). However, they were at the similar level to those determined by Czernych et al. (2017) for the results of the same tests (YES/YAS tests). What is more important, for all controls the values of *R*^2^ were from 0.966 to 0.994 (Table 2), which indicated on the correctness of the performed bioassays.

With regard to the second group of chemicals tested, i.e. the common micropollutants (DCF and EE2), it was found that each of them possessed agonistic activity towards female hormones. It was confirmed by both criteria used in this study. The EC50 values determined for EE2 and DCF were, respectively, four and six orders of magnitude, higher compared to the value of EC50 obtained for the E2 control, i.e. 2.12·10^−10^ M (Table 2). It indicated that EE2 and DCF were significantly less potent to activate the estrogen receptor than E2. Also the results shown in Table 3 confirmed that DCF and EE2 were able to activate the estrogen receptor, and thus have been classified as xenoestrogens. EE2 is a synthetic estrogen hormone and its hormonal potency was many times verified in the estrogenic/androgenic bioassays in order to check the correctness and/or to optimize these assays (Ternes et al. 1999; Bistan et al. 2012).

### Antiestrogenic and antiandrogenic activities

Regarding the antagonistic (inhibiting) activities of the compounds tested to the hormone receptors, the following observations were made.

With respect to the antiestrogenic activity the EC50 values were obtained for two SQAS, DCF and EE2 (Table 2). Among the SQAS, SQAS1 and SQAS2 revealed antiestrogenic potential, however it was significantly lower (three and four orders of magnitude, respectively) than that determined for the 4-HT control, for which the EC50 value was equal to 0.0692·M. It was also lower than the antiestrogenic activity found for DCF and EE2 (Table 2). It should be noticed that the EC50 values for SQAS1 and SQAS2 were extrapolated and their antiestrogenic potency was not confirmed by the IR criterion (Table 3). At the same time DCF and EE2 revealed antiestrogenic activity but at the highest tested concentration tested. Nevertheless, it indicated that they can interact both as agonists and antagonists with the human estrogen receptor.

The study of the antiandrogenic activity, in particular the EC50 values, demonstrated that three out of six compounds studied exerted significant inhibitory effect comparable or exceeding the inhibitory activity of the positive control, i.e. FL. It was clearly observed for SQAS3, DCF and EE2 (Table 2). In the case of SQAS1 and SQAS2, the EC50 values were respectively one and two orders of magnitude higher than that determined for FL (EC50 = 6.78·10^−6^ M for FL) indicating a much weaker inhibitory effect compared to FL (Table 2). At the same time the strongest inhibitory properties were found for SQAS3 and DCF. The EC50 values determined for these compounds were respectively 10-fold and 100-fold, lower than the EC50 value calculated for FL (Table 2). The antiandrogenic potency of SQAS3 and DCF was also confirmed by the IR criterion (Table 3). Regarding SQAS4 it was found that this compound revealed no antiandrogenic activity according to both criteria used (Tables 2 and 3).

EE2 occurred to be an antagonist of androgens as well (Tables 2 and 3). The observations are in agreement with the fact that many xenoestrogens demonstrate antiandrogenic activity (Sohoni and Sumpter 1998). DCF and some of its metabolites were regarded as estrogenic and antiandrogenic compounds (Klopčič et al. 2018). In this work estrogenic activity of EE2 and DCF was primarily reported. Nevertheless, it occurred that they can interact both as agonists and antagonists with the human estrogen/androgen receptors. This study proves that DCF and EE2 belong to hormone-mimicking chemicals of multiple hormonal activities.

### Toxicities towards yeast cells and *Escherichia coli*

**Fig. 1:**
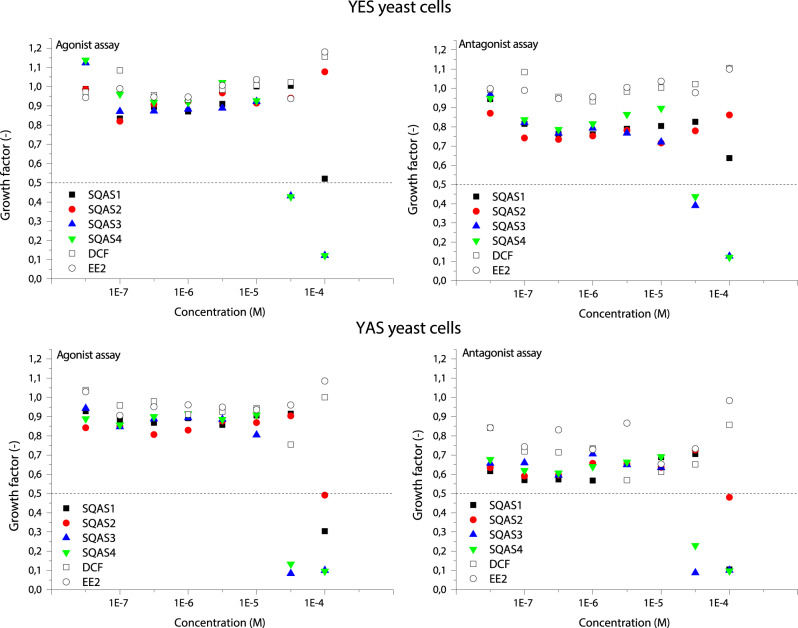
The values of growth factor for YES yeast cells (top panels) and YAS yeast cells (bottom panels)

Simultaneously with the determination of the (anti)estrogenic and (anti)androgenic activity of the compounds tested, their cytotoxicity to the strains of *Saccharomyces cerevisiae* was measured. The values of growth factor for YES and YAS yeast cells are depicted in Fig. 1. Based upon them it was found that all SQAS exerted cytotoxic effects towards cells of any of the genetically modified YES or YAS yeast strains, whereas DCF and EE2 did not show any cytotoxic properties (Fig. 1). SQAS3 and SQAS4 exerted toxic effects towards both YES and YAS yeast cells at two highest concentrations tested (G ≤ 0.5), while SQAS1 and SQAS2 were toxic towards YAS yeast cells only at the highest concentration tested (G ≤ 0.5). It shows that SQAS1 and SQAS2 were less toxic towards the strains of *Saccharomyces cerevisiae* than SQAS3 and SQAS4. The most likely reason of this behavior of SQAS is the acetylation of the hydroxyl groups of the carbohydrate head in the case of SQAS1 and SQAS2 (Table 1). It decreases the cytotoxicity of this class of biosurfactants and contributes to the decrease of their endocrine potency as well.

**Fig. 2:**
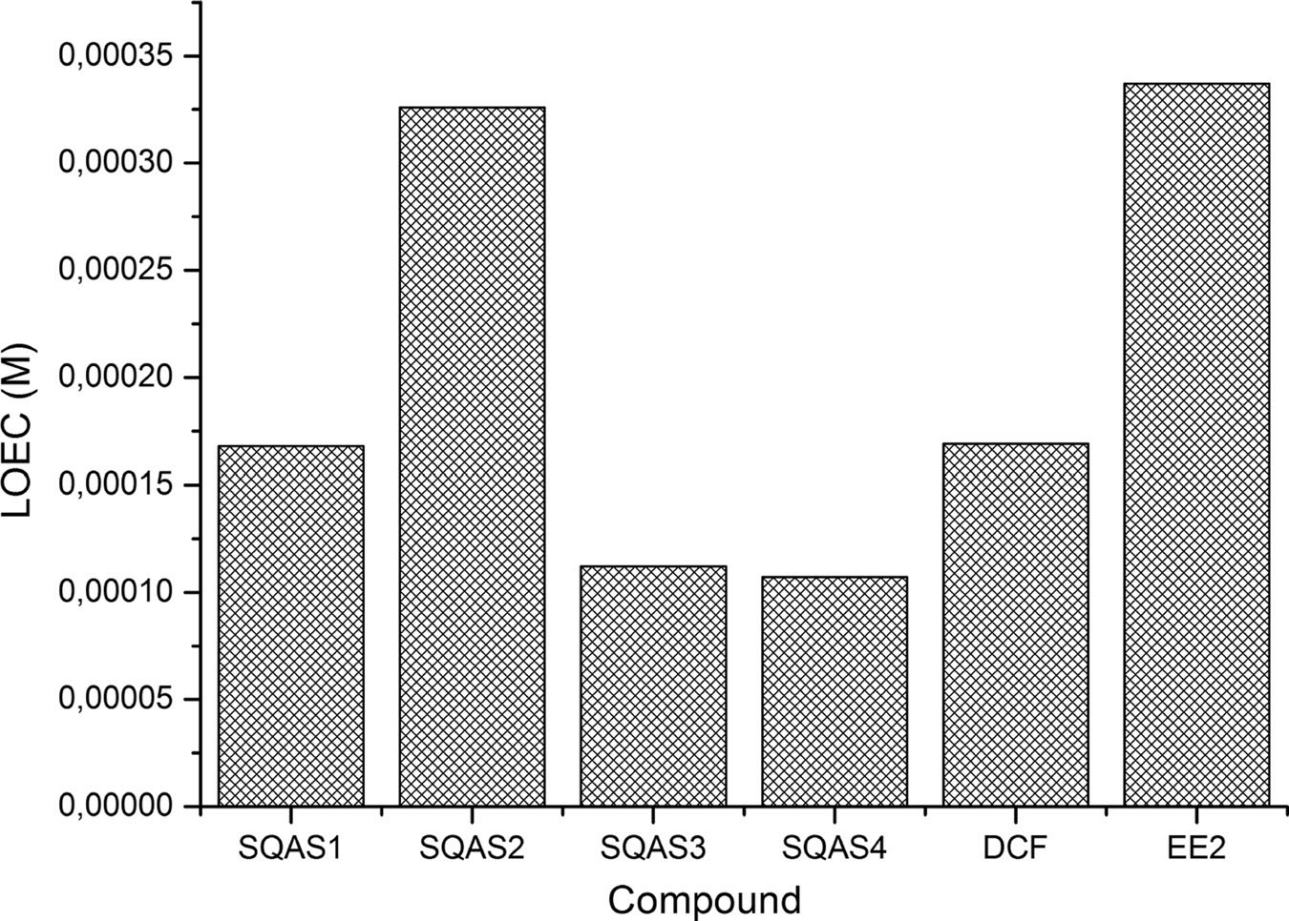
Effect of SQAS, DCF and EE2 on respiration activity of *Escherichia coli*

The toxicity of the studied chemicals towards bacteria *Escherichia coli* was evaluated using the LOEC as estimator. The values of LOEC varied from 1.07·10^−4^ to 3.37·10^−4^ M and despite the difference between them, it should be noticed that all represent the same order of magnitude (Fig. 2). It was found that SQAS3 and SQAS4 might exert a stronger effect on the respiration activity of *E. coli* than the other compounds tested. Regarding the chemical structure of SQAS tested, it occurred that the acetylation of the hydroxyl groups of the carbohydrate head of SQAS caused a decrease of toxicity towards *E. coli* as well. Delbeke et al. (2015) reported that the MIC values for the studied SQAS ranged between 5·10^−6^ and 8·10^−6^ M towards the following bacteria *Bacillus subtilis*, *Staphylococcus aureus*, *Enterococcus faecium* and *Streptococcus pneumoniae*. The lower MIC values indicating a higher toxicity were found for SQAS3 and SQAS4 in comparison to SQAS1 and SQAS2 (Delbeke et al. 2015). The results obtained in this work for SQAS towards *E. coli* are consistent with findings of Delbeke et al. (2015). Out of all biosurfactants studied here SQAS2 turned to be the least toxic followed by SQAS1, SQAS3 and SQAS4 (Fig. 2).

Moreover, it was found that DCF showed relatively strong, actually stronger than EE2, impact on the biochemical activity of bacteria (Fig. 2), while it did not inhibit the growth of the yeasts used in the YES/YAS assays. It proved that the effect of the same compound depended on the sensitivity of the organism which was exposed to it. However, the number of data concerning the effect of SQAS on the organisms is very small and primarily limited to bacteria and yeasts. It makes the evaluation of the possible impacts of this class of biosurfactants on the biota difficult. So before introducing of SQAS to the market, the ecotoxicity studies should be extended and comprise other organisms representing different trophic levels.

## Conclusions

1. None of SQAS studied is most likely an agonist of female or male hormones. SQAS3 shows relatively strong inhibitory activity towards male hormones. SQAS1 and SQAS2 reveal weak antiestrogenic and antiandrogenic potency based upon the values of EC50. Each of these properties is weaker than the potency of the appropriate positive control in the antagonists assays but also compared to the potency of other tested compounds, i.e. DCF and EE2.
2. The unequivocal evaluation of the endocrine potency of the SQAS studied is not an easy task due to their toxic effect at the highest concentrations on the strains of *Saccharomyces cerevisiae* used in the YES/YAS assays. Two out of four SQAS, namely SQAS3 and SQAS4, show cytotoxicity towards both YES and YAS yeast cells at the two highest concentrations tested. SQAS1 and SQAS2 are toxic only towards YAS yeast cells at the highest concentration tested.
3. This work demonstrates that the acetylation of the hydroxyl groups of the carbohydrate head of SQAS decreases the toxicity of these biosurfactants towards *Saccharomyces cerevisiae* and contribute to the decrease of their endocrine potency too.

## Data Availability

Data presented in this study are available on request from the corresponding author.
